# 

**Published:** 1893-03-11

**Authors:** 


					J. /KTOt TVfOKtlNUt. ' 11=1! )E\^ per Ann"11.8s. 6d.. port ftee.
to7. Vol. XIII.?March 11th, 1893. JETI) JUL ?.?tiay part., ea., port n?. m.
Uj'j <ui> aim. mcuuu i mi) m /*> ^
tHJ??3 rt the General Port Office as a Newspaper for W^1 Ditto per Annum; Be., post free.
"Woaiaaion in the United Kingdom and Abroad, j
No. 337. Vol. Xm.] Saturday,
March 11th, 1S93.
[Twopence.

				

